# Ayurvedic management of a chronic venous ulcer using dusting of powdered botanicals – A Case Report

**DOI:** 10.1016/j.jaim.2025.101259

**Published:** 2025-11-08

**Authors:** Arvind Singh Sisodia, Mahesh P. Jadhav, Sanjay C. Babar, Amit Paliwal, Priyanka D. Patil, Manvendra Singh Sisodia

**Affiliations:** a Department of Shalya Tantra, Dr. D. Y. Patil Vidyapeeth, Dr. D. Y. Patil College of Ayurved and Research Centre, Pimpri, Pune, 18, India; b Department of Orthopedic, Shri M. P. Shah Govt. Medical College and Hospital, Jamnagar, 361008, Gujarat, India

**Keywords:** *Avachoornan*, Chronic venous ulcer, Chronic venous insufficiency, *Dushta Vrana*, Topical powdered botanicals, Wound healing

## Abstract

Chronic venous ulcers (CVUs) pose a persistent challenge in clinical practice, especially among elderly individuals with comorbidities such as hypertension and venous insufficiency. Characterised by delayed healing, frequent recurrence, and impaired quality of life, these ulcers often remain unresponsive to conventional treatment. In recent years, Ayurveda has gained attention for its approach to chronic wound care, offering fewer side effects and greater cost-effectiveness.

This report details the case of a 74-year-old male with a history of varicose veins. He presented with a chronic, non-healing venous ulcer over the left lower limb, unresponsive to standard wound management. Diagnosed with a chronic venous ulcer.

The patient was managed with *Avachoornan* (dusting powdered botanicals), composed of equal parts of *Shigru* (*Moringa oleifera*), *Nirgundi* (*Vitex negundo*), and *Guduchi* (*Tinospora cordifolia*) powders. The dressing was applied once daily for four weeks. Progressive improvement in the wound was observed, with noticeable epithelialisation within 1 week.

This case demonstrates the potential of *Avachoornan* (dusting powdered botanicals) as a complementary approach during dressing in managing chronic venous ulcers, warranting further clinical evaluation.

## 1 Introduction

Varicose veins are dilated subcutaneous veins typically measuring ≥3 mm in diameter. These are most commonly observed in the great saphenous vein, the short saphenous vein, and their tributaries [1], which further cause a venous ulcer.

The prevalence of venous leg ulcers is 1.69 % [2]. About 60 % of these ulcers completely healed within 12 weeks. Nonetheless, recurrence is a major issue, with up to 75 % of cases relapsing within three months after initial healing [3].

Conventional management of venous ulcers includes compression therapy, wound care, and surgical interventions. Nevertheless, a considerable proportion of cases recur or evolve into chronic conditions. The average healing period for venous ulcers is approximately 12 weeks, often causing patient discomfort and a diminished quality of life. In this case, however, the healing was achieved in just 4 weeks.

Clinically, venous leg ulcers manifest as cutaneous hyperpigmentation, oedema, prolonged exudation, and delayed epithelial repair—features that correspond closely to *Dushta Vrana* mentioned in Ayurveda. According to *Acharya Sushruta*, the interplay of the vitiated *Tridoshas* along with *Rakta Dushti* impairs *Twak* (skin)*, Mamsa* (muscle)*,* and *Sira* (blood vessels). This leads to *Sira Shithilata* (loss of vessel tone), poor circulation, and stagnation (*Srotorodha*), culminating in ulceration. Clinical features of *Dushta Vrana* [4] include *Atisamvrita* (overly covered), *Ativivrita* (overly uncovered), *Atikathina* (too hard), *Atimrudu* (too soft), *Utsanna* (overly elevated), *Avasanna* (overly depressed), *Atyushna* (calor), and *Atisheeta* (cold to touch) [5], which aligned with the clinical features of this patient.

Ayurveda not only emphasises balancing the body's *Tridoshas* (*Vata, Pitta*, and *Kapha*) using herbal formulations but also the topical application of herbal powders, i.e., *Avachoornan* (dusting powdered botanicals), which facilitates wound healing through multiple mechanisms as it promotes local decontamination, absorbs excess exudate, mitigates inflammation, stimulates granulation tissue formation, and supports re-epithelialisation [4].

## 2 Patient information

• A 74-year-old male with a history of umbilical hernioplasty (2007) and a history of road traffic accident with femur fracture (2014), managed with open reduction and internal fixation and plating, and a known case of hypertension for 1 year on regular medicine (Tab Telmisartan 20mg once a day).
  o History of varicose veins for 6 years with no evidence of deep vein thrombosis as confirmed by a colour doppler study. He has worked as a teacher for the past 10 years. Before that, he was a policeman for 30 years, for which he had a history of prolonged standing.
  o There was no contributory family history. The patient had no history of smoking, tobacco chewing, or alcohol consumption.
• Presenting Complaint: Multiple non-healing ulcers on the left lower leg, associated with pain, itching, burning sensation, swelling, and skin discolouration present over bilateral lower limbs.
• History of present illness: lesions started with itching, and symptoms were insidious in onset and progressive in nature, causing significant discomfort and impaired mobility over a three-month duration.

## 3 Clinical findings

### 3.1 General examination

The patient was conscious, alert, and oriented upon admission. Vital parameters remained within normal physiological limits throughout the hospital stay. Systemic examination findings were unremarkable, with no abnormalities detected across cardiovascular, respiratory, gastrointestinal, or neurological systems.

### 3.2 Local examination (left lower limb)

• According to CEAP {clinical findings (C), etiology (E), anatomy (A), and pathophysiology (P)} classification [6] for varicose venous ulcer, the wound corresponds to C 3,4,6,s E_s_ A_s,p_ P_r,o_.

### 3.3 Inspection

• Skin: The skin over both lower limbs appeared hyperpigmented and thickened, suggestive of chronic venous stasis.
• Swelling: Present in both lower limbs, limiting visual assessment of superficial varicosities.

### 3.4 Venous ulcer

• Ulcer site and shape: Multiple oval-shaped ulcers were observed on the lower medial aspect of the left leg, slightly above the medial malleolus.
• Largest Ulcer Dimensions: Approximately 0.6 cm (length) × 1.1 cm (breadth) × 0.7 cm (depth).
• Ulcer Floor: Covered with thick red granulation tissue.
• Ulcer Edges: Sloping edges observed.
• Skin around wounds: Pigmented with signs of local inflammation and mild oedema.
• Discharge: Serous-purulent discharge noted from the ulcer surface.

### 3.5 Palpation

• Temperature: Localised warmth not appreciable.
• Tenderness: Tenderness around ulcer margins.
• Pedal Oedema: Mild, bilaterally symmetrical oedema present in lower limbs
• Pedal Pulses: Dorsalis pedis, anterior tibial, and posterior tibial pulses are palpable bilaterally, ruling out major arterial insufficiency.

### 3.6 Investigations

**Bilateral lower limb arterial Doppler** (27/12/24) - revealed multiple dilated, tortuous, non-thrombosed varicosities in both lower limbs along the Great and Short Saphenous Veins, with incompetent left Saphenofemoral Junction. Perforators measuring 3.2–3.9 mm on the right and 3.4–3.5 mm on the left were noted in medial and posterior aspects below the knee and above the ankle.

Bilateral Common Iliac Arteries, Superficial Femoral Arteries, Deep Femoral Arteries, Popliteal Arteries, Anterior Tibial Arteries, and Posterior Tibial Arteries show normal flow on colour Doppler with triphasic waveforms.

### 3.7 Diagnostic assessment

The patient has a 6-year history of varicose veins and presents with a non-severe ulcer located over the medial malleolus. The clinical presentation and patient history are consistent with a diagnosis of venous ulcer, with no apparent diagnostic complexity.

According to Ayurveda, based on the patient's symptoms, a diagnosis of *Pitta pradhana* *sarakta Tridoshaja Dushta vrana* was made on his left leg.

### 3.8 Assessment criteria

Assessment of subjective parameters like pain [7], pruritus [8], and burning sensation [9] was done on VAS (Visual Analogue Scale).

All the objective parameters, ulcer floor, edges, exudate, and skin around the wound, were assessed with the Bates-Jensen wound assessment tool [10].

### 3.9 Therapeutic intervention

The patient was on both internal and external medications. (Table 1)

1. Topical Ayurvedic Treatment, i.e., *Avachoornan* (dusting powdered botanicals) with *Guduchi* (Stem) *Choorna*, *Nirgundi* (Leaves) *Choorn*, and *Shigru* (Leaves, Stem Bark) *Choorna* (which are fine powdered and preserved in a glass jar), Mixed in equal proportion and used daily for dressing. Dose - 3 g.
2. Concomitant medication: *Raktashodhak Vati* 500mg BD after food with water for 4 weeks.

**Table 1:** Blood reports.

Blood Reports (dated - 27/12/24)		
1.	Haemoglobin	14.7 gm/dl
2.	RBC	4.88 mill/cu.mm
3.	WBC	5100 cum
4.	Platelet Count	3.01 lac/cu.mm
5.	HIV	Non-reactive
6.	HBsAg	Negative
7.	Bleeding time	2 min 20 sec
8.	Clotting time	4 min 30 sec
9.	PT	15 sec
	INR	1.1
	Control	13.5
10.	HbA1c	5.4 %
11.	BSL (Random)	116 mg/dl
12.	Vitamin B5	200 ng/ml

All these medicines were purchased from the GMP-certified pharmacy of our institution.

### 3.10 Follow-up and outcomes

Daily assessments were systematically recorded during the patient's hospital stay. By the end of the fourth week, the wounds had fully healed, with complete re-epithelialisation observed. (Table 2) However, due to the chronic nature of venous insufficiency, significant haemosiderin deposition was observed, resulting in persistent hyperpigmentation that did not show substantial resolution within the duration of the study. After discharge, follow-up was advised after two weeks, and for the prevention of recurrence, the patient was advised to undergo *Siraveda* (Blood letting therapy) at least once a month, which the patient followed from the panchkarma clinic near his house. First follow-up: Two weeks post-intervention, a reduction in peri-wound skin discolouration was noted, with the skin colour score improving to 3 on the Bates-Jensen Wound Assessment Tool. At the most recent follow-up, this further improved to a score of 2, indicating progressive resolution of surrounding tissue changes.

**Table 2:** Timeline of intervention.

Date	Therapeutic Intervention/Results
27/12/24 Intervention started	Daily dressing (*Avachoornan*) with *Guduchi Choorna, Nirgundi Choorna, and Shigru Choorna* mixed in equal quantities (dressing once a day for 28 days). The fine powders, mixed in equal proportions, were applied to the wound as a topical dressing and packed daily. During subsequent dressings, the wound was cleansed before reapplication. This protocol was followed consistently for four weeks. Internal medication—*Raktashodhak Vati* 500mg BD after food with water for 28 days.
1st Week From 27/12/24 to 03/01/25	Continued the same medication. Result (Table 3)
2nd Week From 03/01/25 to 10/01/25	Continued the same medication. Result (Table 3)
3rd Week From 10/01/25 to 17/01/25	Continued the same medication. Result (Table 3)
4th Week From 17/01/25 to 24/01/25	Continued the same medication. Result (Table 3) (Fig. 1)
1st Follow-up After 2 weeks of completion of the study, 07/02/25	Only wound scars were present. (Fig. 2)
2nd Follow-up After 5 months of completion of the study (June 21, 2025)	Minimal scars marks were present. (Fig. 3)

**Table 3:** Treatment progress.

Parameter for Assessment	Before Intervention	After 1st Week	After 2nd Week	After 3rd Week	After 4th Week	1st Follow-up After 2 weeks of completion of the study, 07/02/25	2nd Follow-up After 5 months of completion of the study (June 21, 2025)
Pain (VAS)	9	8	6	4	2	0	0
Itching (VAS)	8	7	7	2	0	0	0
Burning Sensation (VAS)	7	5	3	1	0	0	0
**BATES-JENSEN WOUND ASSESSMENT TOOL**							
Wound Size (length) × (breadth) × (depth).	0.6 cm 1.1 cm × 0.7 cm	0.5 cm × 1.0 cm × 0.5 cm	0.3 cm × 0.7 cm × 0.3 cm	0.2 cm × 0.6 cm × 0.1 cm	The granulation tissue had covered	Only wound scars were present.	Minimal scars were present.
Depth	3	3	2	2	1	0	0
Edges	4	3	3	2	1	0	0
Exudate amount	3	2	1	1	1	0	0
Skin Colour Surrounding Wound	5	5	4	4	4	3	2

**Fig. 1:**
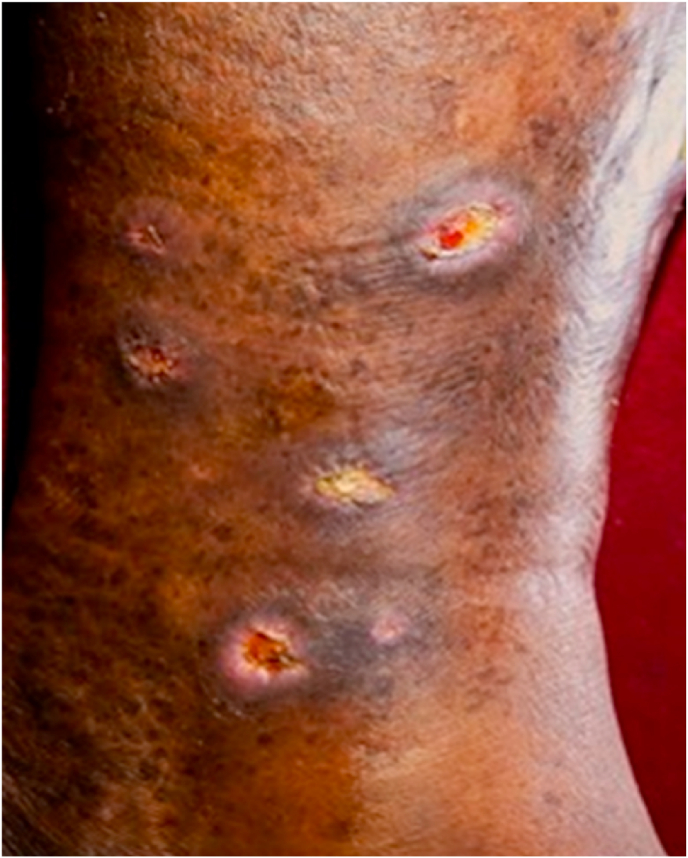
After the 3rd week of Treatment.

**Fig. 2:**
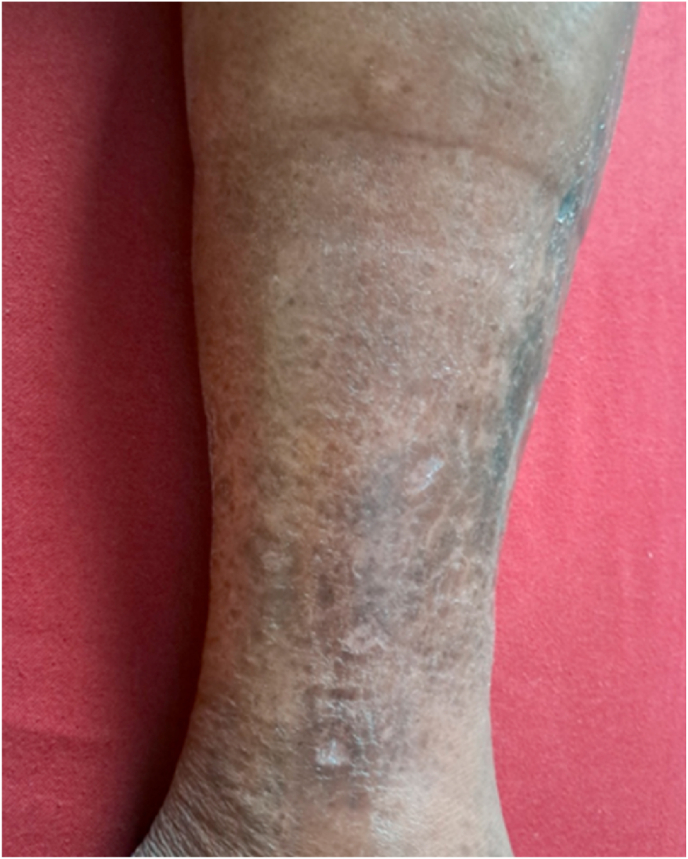
After 2 weeks of completing of study.

**Fig. 3:**
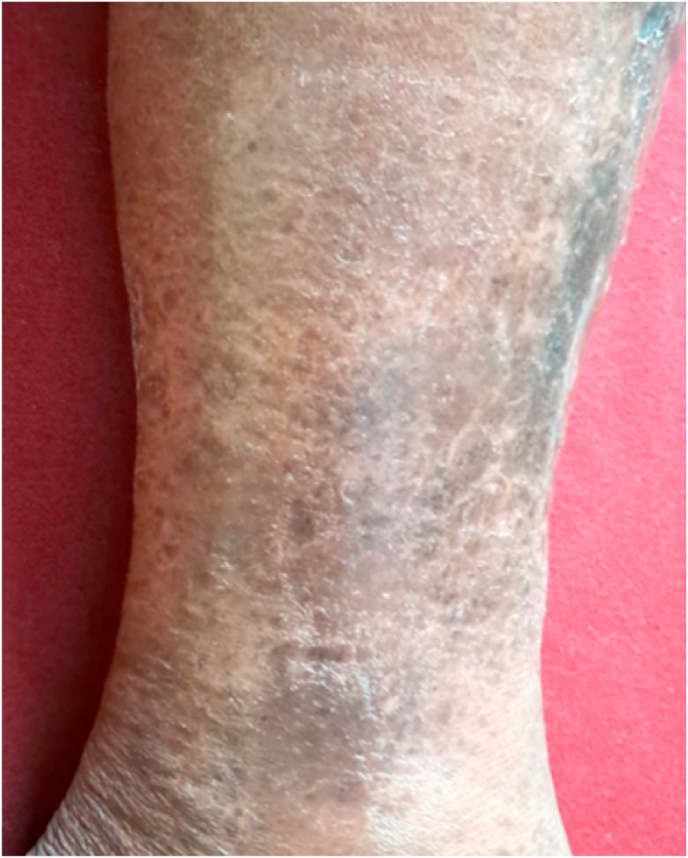
After 5 months (June 21, 2025).

## 4 Discussion

The observed clinical improvements following the *Avachoornan* (dusting powdered botanicals) of *Shigru* (*Moringa oleifera*), *Nirgundi* (*Vitex negundo*), and *Guduchi* (*Tinospora cordifolia*) *Choorna* *and Raktashodhak Vati* in a chronic venous ulcer case underscore the potential therapeutic value of these botanicals in wound management.

Pathophysiology Sustained venous hypertension disrupts physiological haemodynamics, culminating in elevated microcirculatory pressure and capillary dysfunction. This leads to transudation of macromolecules and erythrocytes into the dermal interstitium, accompanied by iron accumulation in the form of haemosiderin [11]. The resultant milieu is characterised by persistent inflammation, oxidative stress, and impaired cellular turnover, all of which impede wound resolution and compromise dermal integrity.

*Shigru* (*Moringa oleifera*) contains flavonoids and phenolics with strong anti-inflammatory and antioxidant effects that reduce oedema and enhance tissue repair by modulating cytokines like TNF-α and interleukins. Its antimicrobial action prevents secondary infections. *Nirgundi* (*Vitex negundo*) offers analgesic and antipruritic effects through iridoid glycosides, supporting comfort and epithelial healing [12]. *Guduchi* (*Tinospora cordifolia*) contributes to *Pitta-Vata* pacification, promotes *Ropana*, and enhances immune responses. Its polysaccharides stimulate macrophage activity, cytokine release, and collagen synthesis, aiding re-epithelialisation, reflecting a classical pharmacological rationale in the management of chronic wounds [13].

*Samprapti* (pathophysiology)- The patient's habitual consumption of *Vidahi* (*Pitta*-aggravating) substances—such as fermented foods, sour soups, *Maṣa* (black gram), prawns, curd, poultry, and refined-flour preparations—contributed to the chronic vitiation of *Pitta* and *Kapha Doshas*. Additionally, his sedentary lifestyle, postprandial sleep, and lack of physical activity further aggravated the *Kapha* and impeded *Agni*, weakening tissue metabolism and clearance. Prolonged exposure to these *Nidanas* led to *Rakta Dushti*, manifesting in tissue toxicity and chronic inflammation. Over time, the chronicity of the condition induced *Vata* vitiation, particularly contributing to pain and delayed healing.

*Samprapti vighatana* (reversal of pathology)- In the *Sushruta Samhita, Avachoornan* (dusting powdered botanicals) is one of the sixty fundamental modalities (*Shashti Upakrama*) employed in the treatment of wounds (*Vrana Chikitsa*). This involves the external application of finely ground herbal powders aimed at promoting wound debridement (*Shodhana*) and facilitating tissue regeneration (*Ropana*) [14]. The choice of *Avachoornan* (dusting powdered botanicals), comprising *Shigru* (*Katu-Tikta Rasa*, *Uṣṇa Virya*, *Kapha-Vata Samaka*), reduced *Shotha* and cleared *Srotorodha*. *Nirgundi* (*Tikta-Katu Rasa*, *Uṣṇa Virya*, *Vata-Kapha Hara*) relieved *Kandu* and *Ruja*, supporting comfort and wound recovery. *Guduci* (*Tshaya Rasa*, *Madhura Vipaka*, *Rasayana*, *Tridosha Samaka*) purified *Rakta*, modulated *Pitta-Vata*, and enhanced *Ropaṇa*. The combination addressed *Dsha-Dshṭi*, facilitated *Sodhana*, *Shamana*, and *Ropana*, aligning with *Sushruta's* principles for managing chronic ulcers. The combination was also selected for its ability to address local Kapha-Pitta aggravation and Vata vitiation.

*Raktashodhak Vati* contains *Anantamoola (Hemidesmus indicus),* which is *Madhura-Tikta rasa, Sheeta virya,* and *Pitta-Vata shamaka* with *Raktaprasadana* properties. *Daruharidra (Berberis aristata)* has *Tikta-Kashaya rasa, Ushna virya,* and acts as *Pitta-shamaka* and *Raktashodhaka. Manjistha (Rubia cordifolia)* is *Tikta-Kashaya rasa, Ushna virya,* and *Raktashodhaka-Varnya. Triphala [Amalaki (Emblica officinalis), Haritaki (Terminalia chebula), Bibhitaki (Terminalia bellirica)]* balances *Tridosha,* enhances *Agnideepana,* and purifies *Srotas. Kutki (Picrorhiza kurroa)* is *Tikta rasa, Sheeta virya,* and pacifies *Pitta* via *Yakrit* and *Ranjaka pitta. Gorakhmundi (Tinospora cordifolia)* is *Tikta-Katu rasa, Ushna virya,* and *Kaphapitta hara. Shuddha Gandhak* (Purified Sulfur) is *Teekshna, Snigdha*, and possesses *Krimighna, Kusthaghna,* and *Raktashodhaka* effects*.* This combination in *Rakshodhak Vati* may have worked on the *nidanas* of the disease.

*Acharya Sushruta* describes *Siravedh* as the foremost intervention for eliminating vitiated *Rakta*, which is central to the pathogenesis of chronic inflammatory disorders. In the present case, *Siravedh* was employed to evacuate stagnant and impure blood, thereby alleviating local *Shotha* (swelling) and *Daha* (burning sensation) and enhancing regional perfusion. This intervention directly targeted *Rakta Duṣṭi*, interrupting the disease process (*Sapti-vighatana*) and establishing a favourable milieu for *Vrana Ropana* (wound healing).

Patient followed our treatment suggestion for prevention of recurrence, following the completion of the study, no ulcer relapse was observed even after five months. (June 21, 2025). (Fig. 3).

Although limited to a single case report, this case study invites further exploration into the integration of Ayurvedic interventions within chronic wound care protocols.

## 5 Conclusion

Chronic non-healing venous ulcers were well managed by the Ayurvedic therapy regimen, which included systemic administration of *Raktashodhak Vati* in addition to dusting of *Shigru* (*Moringa oleifera*), *Nirgundi* (*Vitex negundo*), and *Guduchi* (*Tinospora cordifolia*) powders*.* In situations when traditional treatments might not be sufficient, this case study highlights the potential of Ayurvedic interventions as a comprehensive, secure, and economical method to treat chronic non-healing venous ulcers. In order to create stronger evidence, larger sample-based controlled studies are required.

## Informed consent

Written informed consent was collected from the patient for the publication of this case report.

## Patient perspective

“I had been suffering from recurring ulcers on my left leg for a long time, which severely limited my mobility and comfort. Seeking relief, I came to your Ayurveda hospital—and I'm so grateful I did. The treatment led to a complete recovery in a short span, something I hadn't experienced. Thanks to your care, I've regained my independence and confidence. I continue with the recommended exercises and remain deeply thankful for the healing and support I received.” (June 2025).

## Author contributions

Dr. Arvind Singh Sisodia: Conceptualization, methodology/study design, visual analysis, writing original draft, writing review and editing, Dr. Mahesh P. Jadhav: Software, validation, investigation, visualisation, Dr. Sanjay C. Babar: Resources, data curation, writing review, supervision, project administration, Dr. Amit A. Paliwal: writing review, supervision Dr. Priyanka D. Patil: visualisation, supervision Dr. Manvendra Singh Sisodia: writing review, visualisation.

## Declaration of generative AI in scientific writing

We hereby confirm that the draft of this manuscript is entirely the product of our original work. We affirm that no generative artificial intelligence tools or applications were employed in the original draft of the manuscript's preparation. During the development of the revision of this manuscript, the authors utilised [Copilot] to assist with language refinement and grammar. All content was subsequently reviewed and revised by the authors, who accept full responsibility for the final version of the work. The entirety of the scientific content and conclusions has been developed through our independent expertise and diligent efforts, guided appropriately by our mentor, in strict accordance with the ethical and academic standards prescribed by JAIM.

## Funding sources

None.

## Conflict of interest

The authors declare that they have no known competing financial interests or personal relationships that could have appeared to influence the work reported in this paper.
